# Experiences of husbands of student mothers on a distance learning programme: A phenomenological enquiry

**DOI:** 10.1371/journal.pone.0288779

**Published:** 2023-11-10

**Authors:** Joyce Kwakyewaa Dankyi, Lydia Aframea Dankyi

**Affiliations:** University of Cape Coast, College of Distance Education, Cape Coast, Ghana; University of Nigeria, NIGERIA

## Abstract

Husbands have been the primary support of student mothers in furthering their education. This study aimed to explore the lived experiences of husbands of student mothers (HSM) in a distance education programme of a university in Ghana. Eighteen participants were selected and interviewed. The transcendental phenomenology design was adopted. The sample consisted of all HSM with babies up to 5 years. Coding and content analysis were employed to analyze the data. The study’s findings indicated that husbands experience challenges such as stress, guilt, resentment, and work-family conflict in child care. Coping strategies such as using loan facilities, suspending family projects and using older siblings were adopted by the participants. The study recommends that, Counsellors should orient husbands to understand the need to support their wives and the implications of such support. Husbands should also be educated on basic skills of child care to lessen their frustrations and to avoid stress.

## Introduction

Women’s educational attainment has risen dramatically over the past 20 years throughout the post-industrial world, and female college graduation rates now surpass men’s in some countries [1] even with child care challenges. Women continue to persist in their education since it has been the significant transport tool adopted by society to pass on needed skills and knowledge for economic decision-making by humanity. Support from husbands has been the major reason many of these student mothers persist to complete their educational programmes of study. Traditionally, in most West African countries, it is perceived that the man is the head of the family and breadwinner and therefore needs to be educated for better employment and higher income [2]. The African household has over the years been characterised by patriarchal and hierarchical systems of living [3], with clear and defined roles for men and women in the society [4].

The West African woman, particularly the Ghanian woman, was seen as the heart and keeper of the home that must be trained to keep the kitchen [5]. Women were not formally educated and so could not impact society greatly. However, the African family patterns are slowly but progressively being altered as a result of the process of modernization, education, health opportunities and the ameliorated economic conditions [3]. These factors continuously exert tremendous pressure on contemporary family patterns in the sub-continent, changing the fundamental cultural roles most especially in the domain of child care in the family [6]. Family patterns that were the norm in traditional rural African societies are gradually being altered and substituted by modern values. These changes in the family systems have made it necessary for women to be educated for better employment and higher income. Women now act as co-breadwinners of the family to their male counterparts [7]

The new family trends and patterns have been paralleled by changes in gender roles, especially an expansion of the female role to an economic provider for a family, and lately also the transformation of men’s role with more extensive involvement in family responsibilities, mainly care for children [8, 9]. Child care has been one of the time consuming and attention driven task for women, especially caring for children below age five. Erickson (1978) [10]. is of the view that the first five years of a growing baby is the most critical in terms of attention need and general parental care as personality is developed within this stage. Men and women therefore have to complement each other in child care to ensure the total development of the child for national development since the woman now act as co-breadwinner [7] . Child care within the context of this study is limited to home care such as feeding, changing diapers and soiled clothes, bathing and putting babies to sleep. These activities are the most time demanding and delicate among the other forms.

The inception of distance education, among its benefits, was to provide an opportunity for women who could not leave their families in pursuit of regular educational systems to obtain higher education at the comfort of their homes [11]. Since then, more women have taken advantage of this mode of education in the country under investigation. Distance students experience a lot of challenges as they go through their education because of their characteristics as adults, workers and students. Most of these students have been away from their books for a long time and so adjusting into the learning environment becomes a challenge [12]. Distance education institutions have put in place student support services to ensure successful adjustment and completion of their programmes of study [13].

Student support is critical to the field of education more empirically with matured and distance learning institutions. Among these support services is counselling service [13]. Counselling services help students to overcome some barriers to learning such as time management, finances, child care and concentration. The counselling service is to assist students to adjust to the academic environment, manage their time very well to be able to balance their home, work and the academic environment effectively. Aside this institutional support, family support is also very keen. Support for women’s education through the distance mode has been highly researched both globally and locally over the years, focusing mainly on the family woman and how they cope with their education [14–18]

Support from the family, especially the husband, has also been cited as crucial to the successful completion of the student mother on a distance education programme [14, 15]. As indicated by Mishra, social support plays an important role in academic achievement due to inequalities in education [19]. Husbands as the primary support of student mothers in the course of furthering their education is evident in a study conducted by [15] on student mothers’ struggles and coping strategies in Ghana. The study cited husband’s support as a coping mechanism. They were seen by their wives as their backbone, especially for the successful completion of their programme of study. The main support was in the area of child care and finances.

Other researchers have also indicated that most fathers seem to embrace the idea of active parenting [20–22], especially child care support. This confirms a study conducted about how South African young fathers model masculinity through fatherhood. The analysis reveals that young men deliberately shift their life focus and actively renegotiate their identity through the choice to take responsibility for their children [23]. They structure their personal goals and their relationships with families and partners in terms of providing emotional and financial stability for their children. Thus, fatherhood becomes a highly valorised masculine identity. The study further indicated that, young men resolve the tension between the pursuit of the preservation of men’s domination over women and the traditional gendered roles, and the determination to act as caregivers to their children, thus casting fatherhood as a site to challenge stereotypes of irresponsible young men and absent fathers [23].

Husbands of student mothers supporting their wives to complete their programme successfully may experience some challenges. These challenges may be as a result of the adjustment to the typical traditional roles to take up additional roles as a support to their wives. However, the Social constructionists view knowledge and truth as created and not discovered by the mind [24].The theory allows for a shift from the ideal where they were primarily involved in the family’s economic support, discipline, and control of children to a view that husbands now can play a direct role in the care of their children [25]. Husbands in rendering support especially child care, may experience social identity threat because of gender stereotyping. They may also experience role conflict, and work-family conflict as they combine work with childcare, as well as financial challenges [26]. Husbands in the study are also likely to experience guilt and resentment due to the deeply rooted patriarchal nature of the Ghanaian society [27].

Contextually, the university under study operates the dual mode type of distance education in which students meet physically on campus fortnightly for face-to-face facilitations as well as the administration of examinations. It is purely traditional face-to-face weekend programme with self-directed learning by students during the week days. The face-to- face system puts more demand on the HSM as they struggle to cater for their children on the various campuses. As critical as the surmounting of these challenges by husbands may be to the student mothers’ success, and the total development of the child, it appears most researches especially in the Ghanaian context has neglected it. Therefore, this study will focus on phenomenological inquiry into the experiences of HSM in the University of Cape Coast Distance Education Programme. Specifically, the study will explore the challenges of HSM in rendering support to their wives’ education with regards to child care and finances, and also to find out the coping strategies they adopt to surmount such challenges.

## Research objectives

The study was guided by two objectives which are to:

1. Explore the challenges HSM experience in rendering support to their wives’ education with regards to child care and finances.
2. Find out the coping strategies they adopt to surmount such challenging experiences.

### Theoretical framework

The Social constructionist and the role conflict theories were used to explore the experiences of HSM in child care. The theory of social constructionism was introduced by sociologists Peter L. Berger and Thomas Luckman. The theory states that meaning and knowledge are socially created. Social constructionists believe that things that are generally viewed as natural or normal in society, such as understandings of gender, race, class and disability, are socially constructed, and consequently aren’t an accurate reflection of reality [28]. The constructionist notion of accepting an objective reality implies that HSM can reconstruct their own view of masculinity to and routine roles to adjust to the changes in the family roles.

On the other hand, conflict theory purported by Karl Marx in (1818) [29], states that society is in perpetual conflict because of competition for limited resources. The model describes how HSMs experience role conflict based on their roles as husbands, fathers, and workers [30]. Role conflict happens when there are contradictions between different roles that a person takes on or plays in their everyday life. In some cases, the conflict is as a result of opposing obligations which result in a conflict of interest; in others, when a person has roles that have different statuses, and it also occurs when people disagree about what the responsibilities for a particular role should be, whether in the personal or professional realms [31]. Again, a role conflict is born from the simultaneous occurrence of two or more role requirements, so that performance of one of them makes the performance of the other more difficult [32]. This idea is also used by Fisher [33] who even stresses the impossibility of not fulfilling one of the requirements.

Olorunfemi [34] also postulated that, work-family conflict could happen in three categories: One, Time-based conflict: A situation where the time allotted to work duties hinders workers’ output in a different role (family duties). Husbands in the study may experience time- based conflict as they commit many resources into their various occupations. Two, Strain-based conflict: A situation when stress associated with a particular role (work duties) is transferred to another role (family duties), thereby impeding performance in the latter role. This conflict may be due to stressed up from work, based on the nature of their occupations. The desire to help in child care might be there but because of the stress experienced from the work place, effective child care by the husbands may be affected, Three Behaviour-based conflict: This occurs when a suitably productive behaviour in a particular role (work duties) is unsuitably appropriated in another role (family duties), hence, reducing one’s productivity in the dual role. Based on these premises, the experiences of HSM in child care were explored in the study.

## Research methods

### Study population

The target population for the study consisted of HSM with babies up to 5 years in the University of Cape Coast Distance Education programme. Studies have shown that the first five years of a growing baby are the most critical in terms of attention need and general parental care, (Erickson, 1982) [35]. The focus of the study was on distance education students because of their peculiar characteristics of combining both academic work and family life. The University of Cape Coast was also chosen because it is the leading institution in the provision of Distance Education in Ghana [13]. The nature of the operations of the university demands that students are present every weekend (Saturday and Sunday) for either face to face facilitation or examination. The accessible population was HSM in the Central Region pursuing distance education programme. The distance education students in the central region were chosen because anecdotal records from the counselling unit indicated a more significant number of student mothers using the distance mode from the central region considering either deferment or redrawing from their programmes of study due to lack of understanding and cooperation on the part of their husbands. Most of the issues raised by the student mothers were in relation to child care and finances.

### Sampling procedure

The purposive sampling technique was used for the study. The nature of the study informed the choice of the purposive sampling as the study focuses on HSM with babies up to 5 years in the University of Cape Coast Distance Education Programme. The College operates the study centre approach. That is as part of the support services rendered to students for the successful completion of their programmes of study, face-to-face tutorials are organised fourth nightly to facilitate the understanding of the concepts in the course areas. This is done at various studies centres. Each study centre was considered a stratum for the study. In all, 13 study centres were operational at the time of the study in the central region of Ghana; thus, 13 strata were adopted for the study. All husbands to student mothers who had children up to 5 years were purposively selected from each stratum (study centre).

The sample size for the study was 18 at a saturation point since the emphasis was on comprehensiveness and not necessarily on numbers. This was representative enough as HSM present homogeneous characteristics; increasing the population size would not have added new information but just increase the numbers [36].

### Data collection

In the data collection process, the researchers relied on the assistance of study centre coordinators in identifying all student mothers with babies up to 5 years. The identified HSM were contacted through face-to-face interactions and on phone calls to seek their informed consent to be part of the study. The Consolidated criteria for reporting qualitative research (COREQ) by [37] was followed. The researchers paid a first time visit to establish a good relationship with the participants prior to the data collection. Video calls were also used to establish a relationship with husbands who were not at the study centres. All participants were informed about the purpose of the research and the objectives it sought to achieve. The interviews were single time, and only interviewers and interviewees were around at the time of the interview.

Two researchers conducted all the interviews with field notes alongside the interview. All the two researchers were females with Ph.Ds. and had a qualitative research background. A self-developed interview guide informed by the study’s objectives and fine-tuned by experts’ opinions in the area of the study was used. Based on the specific objectives of the study, three themes were carved to construct the interview guide. The interview guide ensured open discussion to ascertain detailed information on the issues. The first theme focused on challenging child care experiences, while the second and third themes focused on challenging financial experiences and coping strategies. The interviews were audio-recorded. The interview duration was between 30 to 60 minutes, with the date and time being at the convenience of the participants.

The phenomenological design which tries to explain why and how there is a relationship between two aspects of a phenomenon or situation [38, 39] was adopted for the study. Specifically, the transcendental phenomenology design was employed for the study. The design helped the researchers to hear the account of the HSMs as they rendered child care and financial support. Again, the adoption of transcendental phenomenological design for the study enabled in-depth information to be unearthed from the study’s participants [40, 41].

To ensure a fair representation of the participants, the researchers interviewed one husband each from the 13 study centres during the first round. The interview continued until themes within the data collected were repeated within the period of the interview map activity till no themes and codes appeared to emerge. COVID- 19 protocols such as washing of hands, wearing of nose mask, social distancing and sanitation of hands and surfaces were adhered to. Thus, the sample consisted of 18 husbands from 13 study centres. Table 1 shows the participants’ demographics.

**Table 1:** Participants characteristics.

Pseudo Name	Age	No. of Children (0-5yrs)	Occupation	Employment status
Ean	50–59	1	Shop attendant	Full time
Tan	40–49	1	Civil servant	Full time
Gan	40–49	1	Teacher	Full time
San	30–39	1	Doctor	Full time
Yan	40–49	2	Teacher	Full time
Man	40–49	2	Nurse	Full time
Zan	30–39	1	Hospital Administrator	Full time
Ban	30–39	2	Trader	Full time
Pan	50–59	1	Entrepreneur	Full time
Van	40–49	2	Banker	Full time
Kan	40–49	1	Surveyor	Full time
Gan	40–49	2	Driver	Full time
Aan	40–49	1	Maison	Full time
Fan	30–39	2	Security	Full time
Han	30–39	1	Architect	Full time
Jan	40–49	2	School Administrator	Full time
Ran	40–49	1	Teacher	Full time
Qan	50–59	2	Farmer	Full time

### Data analysis

We uploaded all responses to the open-ended question into Microsoft Excel. The coding team were the two researchers both with Ph.D. in Guidance and counselling and qualitative research background. We coded all data using inductive approaches. First, we read all responses to generate preliminary, inductive codes. We then met to develop the codebook by explicating definitions, inclusion criteria, and exclusion criteria for each code. Using this codebook, we independently coded the first 20 open-ended responses and met to discuss discrepancies and made necessary modifications to the codebook. Once the codebook was finalized, we independently coded the entire set of responses. We used Merriam and Tisdell [42] process of analytical coding. Then, we discussed any discrepancies in our coding to reach consensus and generated the final coded datafile. Finally, we met to discuss relationships between codes to conceptualize broad themes using thematic analysis. We used reflexivity throughout the coding to avoid our own experiences and identities influencing our interpretations of the data.

### Quality assurance

To establish the qualitative data trustworthiness and authenticity, Lincoln and Guba, (1985) [43]. criteria for demonstrating rigour within qualitative research, namely true value, consistency and neutrality, and applicability, were adhered to. On true value, informed consent was sought from all participants, and their willingness to share their experiences in-depth and overtime enabled clarification of findings. The audio-recorded interviews allowed for a repeated revisiting of the data to check emerging themes and remain true to participants’ accounts of their views on the experiences of HSM. Rich and thick verbatim extracts from participants were reported. This will assist readers in making judgements about whether the final themes were true to participants’ accounts.

Participants were also invited to comment on the research findings and themes. In the case of consistency and neutrality, a transparent and precise description of the research process was ensured at all study stages. A research diary was also kept in documenting challenges and issues, assisting in maintaining cohesion between the study’s aim, design and methods. Emerging themes were discussed by the researchers who had qualitative research expertise in an open process where assumptions were challenged, and a consensus was reached. In terms of applicability, rich detail of context, conclusions and recommendations was provided to ensure the transferability of the study to other regions in the country.

### Ethical consideration

Ethics is an essential consideration in research that involves human subjects [44]. Ethical approval was obtained from the Institutional Review Board (UCCIRB/EST/2020/40) of the University of Cape Coast, Ghana. Data management concerning this study was considered in terms of the storage of the data collected. The recorded data were kept under secured password protected device. In the study, ethical issues considered were the right to privacy, voluntary participation, no harm to participants, confidentiality, no deception, and no scientific misconduct. In addressing issues of informed consent, information which describes the benefits, risks and procedures for the research was read and explained to the participants and they were also given an opportunity to ask any question about the research. The participants were made to sign their informed consent and to also ensure voluntary participation.

The purpose of the study was explained to the participants and they were assured that all information they provide will be used purely for academic purposes and treated with utmost confidentiality and anonymity. They were also assured that; their responses were not in any way going to affect the academic grading of their wives. It was also emphasized that their wives never reported them as being unsupportive. The respondents were further assured of keeping their information shared as confidential as possible. For anonymity’s sake, the participants were not required to mention their names or any information that personally identifies them during the interview and the report was done with pseudo names.

## Results

By listening to the participants’ narratives, we discerned several challenging experiences attributed to participants support to their wives’ education. These experiences were in the areas of child care and finances. The identified themes describe: 1) child care challenging experiences such as stress, guilt, resentment, and work family conflict, 2) financial challenging experiences such as inability to pay school fees, inability to start or continue a family project and undue pressure on the family’s finances, and 3) Coping strategies such as the use of loan facilities, suspending family project, using older siblings, using extended family members, hiring a nanny and weekend school.

### Child care challenging experiences

Participants expressed child care challenging experiences as guilt and resentment, work family conflict and stress. These experiences of some participants were due to time and attention demands of child care, changing of diapers and the washing of soiled cloth. Some participants experienced guilt and resentment as they changed diapers and wash babies’ soiled clothes. The men had the desire to take up their new roles in child care but because they have not been able to construct their own meaning, they experienced guilt because of societal demands. This is evidence in the excerpts below:

One participant said that: I’ *ve just felt this overwhelming sense of guilt like*, *am I less of a father*, *am I less of a man*, *have I changed my identity as a man for changing diapers and even putting the baby to sleep*? *Anyway*, *sometimes I also feel you can still be a man and still support in child care (Tan)*.

Another participant narrated his experience with child care and embarrassment he had to endure by stating that: *I have never been so humiliated in my life like this before*, *when a friend called me "Kwadwo Besia”(literally translated*, *man- woman)*, *all because I was changing my 1 year old baby’s diaper when the mother had left to write an exams at their study centre*. *The most shameful part was when I went to work the following morning and it was the talk of the day*. *Since then*, *I have been a little sceptical to perform such role*.

The interviewers also indicated they experienced role conflict as a result of combining child care and work. This leads to work family conflict which may be partly due to lack of priority of goals. Gab confirmed this by saying that: ‘*Being a caring father and committing to my job as a career person are intertwine with the core of my being*. *It is just sometimes difficult to reconcile both being a caring father and committing to my job*. *I find conflict and tension*. *Spending sufficient time and attention on my work place and also with my kids do not seem possible as a medical doctor and husband of a student mother*. *I am still trying to figure out how these two parts of me can interact better*.

Surprisingly, some participants also demonstrated some inappropriate behaviours as they combine child care and work. A participant narrated: *I sometimes have to pretend not to be fit for work just to take care of the last born who is a bit difficult to handle by the mother together with her studies whiles she prepares for her end of semester exams*. *I feel so guilty any time I have to abandon my work duties*. *Oh*! *How I wish I have control over this (Van)*

Stress was also one of the challenging experiences the participants went through in their involvement with child care. Putting babies to sleep whiles controlling for cries and feeding the baby brought a lot of stress to most participants. San opined that: *I never imagined myself in this whole business of childcare when I was single till my son came to the seen*. *Women are blessed with special grace when it comes to childcare because controlling for crying and putting the baby to sleep is a whole profession on its own*. *I become so frustrated and worried when I had tried all means and yet the baby would still be crying (San)*.

Similarly, Pan said that: *Feeding the baby initially was hell and very stressful for me*. *My wife expressed breast milk for me to warm for the baby only to end up hurting the baby because it was too hot for the baby*. *I had no idea about the process involved in going about the feeding of the baby (Pan)*.

Work family conflict and stress were also expressed by participants in terms of Time-based, Strain-based and Behaviour-based conflict. These are evidence in the excerpts below:

On the issue of time-based stress, a participant had this to say: *I sometimes have to go to work late*, *in a few instances I have had to answer queries for this kind of attitude towards work*. *As I told you earlier on*, *my family is first and so I do a lot of sacrifices for the family (Sam)*.

Again, on the issues of strain-based stress, a participant narrated that, *to be honest*, *it is very stressful and demanding in caring for children at this level*. *Almost every day I have to push some activity at the work place aside in other to be at home early to assist in childcare (Van)*.

Furthermore, a participant said *that*: *I do experience a lot of work family conflict and it comes most of the time*. *There are instances where I have to go to work late and even sometimes do not go at all because I have to travel with my wife from Mankesim to Cape Coast for her to write her exams*. *(kan)*.

### Financial challenging experiences

Financial challenges such as participants’ inability to pay school fees, inability to start or continue a family project and undue pressure on the family’s finances were the major cause of financial burdens to participants. Tan, fan and Gan expressed this in the excerpts below:

The interviewers indicated the financial challenges were due to the payment of school fees and other family commitments. They have to sacrifice some family projects in other to pay for their wives’ school fees and other expenses. Some of the direct statements of the participants are quoted below:

One participant narrated that: *I have to pay the children’s fees alone*, *but before the programme*, *she used to support in paying the fees but since she is now paying her own fees for the programme*, *I have to do that alone*. *I have even paused a building project we started because of the extra financial burden brought by her schooling*. *She has even gone ahead to bring her sister to support her in terms of caring for the baby*. *Although i don’t pay her fees I have to increase the house keeping money because of the additional person in the house (Can)*.

Another participant passionately said that: *Hmm*, *as for financial issues they are enormous*. *Paying school fees*, *renting a guest house and taken care of the home*. *I feel extra load has been added to the already loaded load*. *We have suspended our building project just to pay for my wife’s school fees*. *Last semester we went for loan before we were able to survive for the month (Gan)*.

Interestingly, the participants attributed the financial hardships to lack of planning on the part of the family. A participant had this to say: *Her schooling has brought a lot of pressure on the family finances*. *I have to pay her fees*, *accommodation and the nanny taken care of the baby*. *I saw this initially when she decided to start the programme and therefore advise her to wait but you know women*, *she did not listen and now we have to experience a lot of pressure on our finances (Aan)*.

In addition, Fan had this to say: *I am a security man and therefore would always be at work*. *I have to combine my work as a security man and the caring of the 2kids who are all under 5years for my wife to be able to write her exams since I don’t earn much to afford a house help*. *Her education has actually brought about a lot of burdens on our finances*. *I am only hoping things get better when she completes (Fan)*.

### Coping strategies

Coping strategies, such as the use of loan facilities, suspending family project, using older siblings, using extended family members, hiring a nanny and weekend school, were used by participants. The assistance of extended family, members such as mother in-laws was solicited. This sometimes brought extra financial burden, and other family conflicts among the family members. Evidence in the following excerpts.

A participant reported that: *My work is so demanding that I hardly take care of the child myself when my wife goes for lecture*. *I normally get help from my mother in-law*, *who sometimes bring in certain practices that may not help in the development of the child’s personality (Zan)*.

Another participant confirmed a similar experience by stating that: *Sometimes in an attempt to put the baby to sleep or stop crying*, *she ends up throwing the baby up and down so high which can affect the baby’s brains but what can I do*? *i have no option than to keep quiet (Zan)*.

Some participants also resorted to borrowing of money from banks and friends. Excessive borrowing also led to financial hardships in the family. Others had to suspend ongoing projects in the family to support their wife’s education. A participant had this to say: *I borrow money from friends and sometimes soft loans from my welfare contributions before we are able to pay both her fees and that of the weekend school*, *we have enrolled the children (Han)*.

Another participant added that: *As an entrepreneur I started a small-scale business which I needed to commit money every month towards it for its sustainability*, *but I have to suspend it in other to see my wife through her education and also care for the child (Pan)*.

Surprisingly, some participants resulted to sacrifice their older siblings to give support to the student mother. These older siblings have to abandon school in other to take care of their younger siblings. Jan had this to say: *My first born who is currently in JHS2 has been very supportive in taken care of her younger sister*. *Sometimes she has to sacrifice her Friday’s attending school to accompany the mother to her study centre since they have to travel on the Friday*. *I only pray that this will not affect her academic work (Jan)*.

## Discussion

The theoretical conceptions and the literature for the study were helpful in exploring the experiences of HSM in relation to child care and financial support. The role conflict theory helped to deepen understanding of the stress, guilt, resentment and work family conflict that the husbands in the study go through. The husbands experience these challenges when they find themselves pulled in various directions as they try to maintain the many statuses they hold as husbands to student mothers, fathers and career men [30]. The social constructionist theory also helped to understand how HSM can reconstruct their own realities based on their new roles to challenge gender stereotypes [18]. The discussions are done in line with the three main themes identified during the analysis stage (child care experiences, financial experiences and copping strategies).

On the issue of child care experiences, the results indicated that most of the husbands in the study experienced stress as they combined both child care and career related jobs. This may be due to time and attention demand by children up to five years [45]. This confirms why a participant said, it *was* very stressful and demanding in caring for children at that level.. The first five years of life of the child are a period of profound growth and changes as children begin to speak, think, reason, and feel and therefore need for a responsive caretaker. Yucel, Şirin [46] also added that role conflict also occurs when individual experience incompatible work demands which intern led to stress.

The husbands also experienced guilt and resentment because of the negative comments by colleagues. This is not surprising because the Ghanaian society is seen as deeply patriarchal [27] and therefore there are distinct gender roles. The participants seem not to have constructed their own reality when it comes to child care as indicated by Tan, the overwhelming sense of guilt *he* had felt as a result of , changing diapers and putting the baby to sleep They also go through this because of their inability to combine both roles as in childcare and career related jobs which can sometimes lead to undesirable behaviour such as older sibling sacrificing their education to take care of younger siblings as evident in the study. This is in line with the assertion that behaviour base conflict occurs when suitably productive behaviour in a particular role (work duties) is unsuitably appropriated in another role (family duties), hence, reducing one’s productivity in the second role [32, 47]

Again, the findings of the study revealed that, husbands experienced financial challenges such as their inability to pay school fees, inability to start or continue a family project and undue pressure on the family’s finances. These financial challenges caused stress and guilt to husbands. This is not surprising because in the Ghanian society men are expected to take up all financial responsibility. Failure to execute all financial obligations threatened their very existence as men. This is where the counsellors role to help men construct their own view of being a good father in the face of modernization is very crucial [48] . No wonder some of the participant indicated they have to pay fees for both the student mother and their children. The challenges such as inability to pay school fees, start or continue a family project are the typical masculinity role of fathers that needs reconstruction according to [24] .This reconstruction is only possible with the help of a counsellor.

With regard to coping strategies, participants indicated the use of loan facilities, suspending family project, using older siblings, using extended family members, hiring a nanny and weekend schools as coping strategies adopted. The use of extended family members as part of the copping strategies used by the participants is good and purely Ghanaian. It is also a key strategy as Social factors have been found to play an important role in academic achievement [19]. However, using older siblings to support younger siblings sometimes comes as a form of child abuse. As was revealed in the study, older siblings had to abandon their school in other to attend to their younger siblings. The idea of these older siblings sacrificing their education is regarded as child abuse and totally unacceptable.

On the other hand, loan facilities as coping strategies by the husbands, can bring a lot of stress and hardships into the family if not well planned, which may affect the peace and love at home [49]. Research has also indicated that borrowers have a high probability of experiencing excessive financial hardship in repaying loans when their incomes are at the low end of the distribution of incomes [49]. There is therefore the need for counsellors to assist HSM to understand themselves and their environment so as to make the best out of the many strategies available to them.

## Conclusions

In sum, the support from husbands has been the major reason many of the student mothers persist to complete their programmes of study. Husbands in supporting these student mothers have suffered from external pressure and negative repercussions associated with gender roles from society. This has led to the feeling of guilt and resentment by these husbands. Men also experience work family conflict due to the many roles they have to perform based on the many statuses assigned to them by society which sometimes lead to stress. Participants adopted coping strategies, such as the use of loan facilities, suspending family project, using older siblings, using extended family members, hiring a nanny and weekend schools. Husbands therefore need to be oriented and counselled to transform how they think about child care to avoid guilt and resentment. This is possible when husbands understand the need and the implications of their support in child care.

### Recommendations

Based on the key findings and conclusions from the study, the following recommendations are made for policy and programme interventions in the university under study.

First, it is recommended to the Ministry of Gender, Children and Social Protection to review their existing models of support towards female mothers’ education. The ministry can incorporate strategies such as education and orientation on child care for husbands as a way of equipping husbands to successfully assist their wives to navigate their educational programmes.

Secondly, it is also recommended to the Management of the College of Distance Education of the University of Cape Coast to establish Child relaxation rooms in each of its study centres. This could serve as waiting rooms to husbands who accompany their wives to the various study centres. Management of the College of Distance Education can consider their mode of operation by shifting to blended learning or fully online learning.

Again, the Management of the College of Distance Education Should laisse with NGOs to establish a well-resourced weekend schools or day care centres to take care of children for husbands to perform other roles to avoid role conflict.

Furthermore, Counsellors of the College should counsel husbands to understand the need to support their wives and the implications of such support. Husbands should also be educated on basic skills of child care to lessen their frustrations and avoid stress when rendering child care support.

## Supporting information

S1 Dataset (DOCX)
