# Supplementary material for: Experiences of husbands of student mothers on a distance learning programme: A phenomenological enquiry
Source: PLoS One. 2023 Nov 10;18(11):e0288779. doi: 10.1371/journal.pone.0288779 (PMC10637649; doi:10.1371/journal.pone.0288779)
Supplement: S1 Dataset — (DOCX) [file pone.0288779.s001.docx]

DATA FOR THE MANUSCRIPT (TRANSCRIPTS OF THE PARTICIPANT)

**UNIVERSITY OF CAPE COAST**

**COLLEGE OF DISTANCE EDUCATION**

**INTERVIEW GUIDE FOR HUSBANDS OF STUDENT MOTHERS**

**TOPIC: PHENOMENOLOGICAL ENQUIRY INTO THE EXPERIENCES OF HUSBANDS OF STUDENT MOTHERS IN THE UNIVERSITY OF CAPE COAST DISTANCE EDUCATION PROGRAMME**

Dear Sir,

*We are conducting research on the above topic, and you have been selected as one of the key participants. We would be most grateful if you could participate in the interview. Information obtained from this interview would be treated with utmost confidentiality. We appreciate your co-operation in advance for taking part in the study.*

**THEME ONE: Challenging Experiences with Finance**

1. What are the financial challenging experiences you go through in course of supporting your wife’s education? Probe for issues such as:
2. How challenging is it to support your wife to pay fees
3. financial commitment to the family as a husband
4. How does your support as a husband put pressure on the finances of the family (Probe for hiring a nanny, transportation to and fro the study centre, baby sitter and accommodation at the study centre )

**THEME TWO: Challenging Experiences with Childcare**

1. How stressful is child care as a support to your wife**?** Probe for issues such as**:**
2. Time and attention to the child
3. Changing diapers
4. Putting babies to sleep whiles controlling for cries
5. Feeding the baby
6. Do you experience work family conflict because of your support as a husband? Probe for issues such as**:**
7. Time and attention
8. conflict between work and child care
9. How does your support as a husband lead to social identity treat? Probe for issues such as:
10. Guilt and resentment

**THEME THREE: Coping Strategies**

1. How do you cope with the financial challenges associated with supporting your wife’s education? Probe for issues such as**:**
2. Loans
3. Borrowing from others
4. Suspending other financial activities
5. How do you cope with childcare challenges associated with the support you give to your wife’s education? Probe for issues such as**:**
6. Hiring a nanny
7. Seeking assistance from family members
8. Enrolling the child in school
9. Using older siblings

**Childcare challenging experiences**

I' *ve just felt this overwhelming sense of guilt like, am I less of a father, am I less of a man, have I changed my identity as a man for changing diapers and even putting the baby to sleep? Anyway, sometimes I also feel you can still be a man and still support in child care (Tan).*

*Being a caring father and committing to my job as a career person are intertwine with the core of my being. It is just sometimes difficult to reconcile both being a caring father and committing to my job. I find conflict, tension, and guilt within myself as I find it difficult to practise both. Spending sufficient time and attention on my work place and also with my kids do not seem possible as a medical doctor and husband of a student mother. I am still trying to figure out how these two parts of me can interact better (Gab)*

*I sometimes have to pretend not to be fit for work just to take care of the last born who is a bit difficult to handle by the mother together with her studies whiles she prepares for her end of semester exams. I feel so guilty any time I have to abandon my work duties to take care the child. Oh! how I wish I have control over this (Van)*

*I have never been so humiliated in my life like this before, when a friend called me "Kwadwo Besia”(literally translated, man- woman), all because I was changing my 1 year old baby's diaper when the mother had left to write an exams at their study centre. The most shameful part was when I went to work the following morning and it was the talk of the day. Since then, I have been a little sceptical to perform such role especially when people are around even though my decision has affected my wife.*

*:*

*I feel so guilty sometimes for taken up some child care support especially with children below 5 years old. I have to go the extra mile of washing soiled clothes and even bathing my girl. I have received a lot of criticism from my family members especially my mother and sister and this sometimes makes me feel awkward and wish to stop, looking for an alternative way of getting a nanny (Yan).*

*My recent experience that made me feel so guilty and wanted to stop washing the baby’s clothes, was when my colleagues gathered and talked about how I have been turned into a woman in my own home by my wife (Ban).*

Putting babies to sleep whiles controlling for cries and feeding the baby brought a lot of stress to most participants. San and Pan had this to say:

*I never imagined myself in this whole business of childcare when I was single till my son came to the seen. Women are blessed with special grace when it comes to childcare because controlling for crying and putting the baby to sleep is a whole profession on its own. I become so frustrated and worried when I had tried all means and yet the baby would still be crying.*

*Although I experience guilt because of the comments I received from both my family members and colleagues, I still perform that role because of the benefit of her education to the family (San).*

*Feeding the baby initially was hell for me. My wife expressed breast milk for me to warm for the baby only to end up hurting the baby because it was too hot for the baby. I had no idea about the process involved in going about the feeding of the baby (Pan).*

Work family conflict and stress were expressed by participants in terms of Time-based, Strain-based and Behaviour-based conflict. These are evidence in the excerpts below:

*I sometimes have to go to work late, in a few instances I have had to answer queries for this kind of attitude towards work. As I told you earlier on, my family is first and so I do a lot of sacrifices for the family at the expense of my work (Sam).*

*To be honest, it is very stressful and demanding in caring for children at this level. Sometimes I become so tied and wish I had an option but I have to endure for the mother to prepare for her exams. Headache and fatigue are constant in combining both childcare and work. Almost every day I have to push some activity at the work place aside in other to be at home early to assist in childcare (Van).*

*I do experience a lot of work family conflict and it comes most of the time. There are instances where I have to go to work late and even sometimes do not go at all because I have to travel with my wife from Mankesim to Cape Coast for her to write her exams. I cannot afford paying someone to do this job, and even I have to stay with her from Friday to Sunday when I should have been to work. Anyway, I can’t complain because of the future benefits (kan).*

**Financial Challenging Experiences**

Financial challenges such as participants’ inability to pay school fees, inability to start or continue a family project and undue pressure on the family’s finances were the major cause of financial burdens to participants. Tan, fan and Gan expressed this in the excerpts below:

*I have to pay the children's fees alone, but before the programme, she used to support in paying the fees but since she is now paying her own fees for the programme, I have to do that alone. I have even paused a building project we started because of the extra financial burden brought by her schooling. She has even gone ahead to bring her sister to support her in terms of caring for the baby. Although i don’t pay her fees I have to increase the house keeping money because of the additional person in the house (Can).*

*Hmm as for financial issues they are enormous. Paying school fees, renting a guest house and taken care of the home. I feel extra load has been added to the already loaded load. We have suspended our building project just to pay for my wife's school fees. Last semester we went for loan before we were able to survive for the month (Gan).*

*Her schooling has brought a lot of pressure on the family finances. I have to pay her fees, accommodation and the nanny taken care of the baby. I saw this initially when she decided to start the programme and therefore advise her to wait but you know women, she did not listen and now we have to experience a lot of pressure on our finances (Aan).*

*I am a security man and therefore would always be at work. I have to combine my work as a security man and the caring of the 2kids who are all under 5years for my wife to be able to write her exams since I don’t earn much to afford a house help. Her education has actually brought about a lot of burdens on our finances. I am only hoping things get better when she completes (Fan).*

**Coping Strategies**

Coping strategies such as the use of loan facilities, suspending family project, using older siblings, using extended family members, hiring a nanny and weekend school were used by participants. This is evidence in the following excerpts.

*My work is so demanding that I hardly take care of the child myself when my wife goes for lecture. I normally get help from my mother in-law, who sometimes bring in certain practices that may not help in the development of the child’s personality. Sometimes in an attempt to put the baby to sleep or stop crying, she ends up throwing the baby up and down so high which can affect the baby’s brains but what can I do? i have no option than to keep quiet (Zan).*

*I borrow money from friends and sometimes soft loans from my welfare contributions before we are able to pay both her fees and that of the weekend school we have enrolled the children (Han).*

*As an entrepreneur I started a small-scale business which I needed to commit money every month towards it for its sustainability, but I have to suspend it in other to see my wife through her education and also care for the child (Pan).*

*My first born who is currently in JHS2 has been very supportive in taken care of her younger sister. Sometimes she has to sacrifice her Friday’s attending school to accompany the mother to her study centre since they have to travel on the Friday. I only pray that this will not affect her academic work (Jan).*
